# Attitudes and awareness of professionally active people on eye diseases prevention—a descriptive cross-sectional survey

**DOI:** 10.3389/fpubh.2025.1553361

**Published:** 2025-06-03

**Authors:** Karina Mierczak, Anna Garus-Pakowska

**Affiliations:** Department of Nutrition and Epidemiology, Medical University of Lodz, Lodz, Poland

**Keywords:** attitudes, awareness, eye diseases, eye prevention, eye protection, vision, vision disorders, vision health

## Abstract

**Introduction:**

The use of screen devices is a common aspect of everyday life. Additionally, for the majority of adult workers, their job is closely associated with using monitors. Prolonged or improper use of screens, especially at a close distance from the eyes, can result in eye fatigue and the development of basic refractive errors. Professional workers are particularly susceptible to the onset of certain vision disorders, notably those related to prolonged near work and screen exposure (such as myopia), and awareness of potential risks contributes to the mitigation of negative consequences associated with visual impairments.

**Objective:**

To assess the level of awareness and attitudes regarding preventive measures for eye diseases among working individuals.

**Material and methods:**

A survey was conducted from September 27th, 2023 to December 1st, 2023, among 251 working individuals. The research tool consisted of a custom proprietary survey questionnaire. The obtained survey results were subjected to statistical analysis using non-parametric tests (χ^2^, Fisher's, Kruskal-Wallis) and parametric tests (Bartlett's, ANOVA), depending on the type of data.

**Results:**

Only 32.27% of participants demonstrated a satisfactory level, and 30.28% a good level of awareness regarding the prevention of eye diseases. A correlation was found between the level of awareness and education. Individuals with intermediate and higher education most frequently received satisfactory and good evaluations. The highest mean score was 25.55 out of 38 possible points and was achieved by participants with higher education. No significant correlation was observed between the level of awareness and the type of occupation.

**Conclusions:**

The level of awareness and attitudes of the surveyed group of working individuals regarding the prevention of eye diseases is limited. Therefore, it is crucial to strive for a change in the current state and to expand the awareness of working individuals in Poland regarding the safe use of screen devices in both stationary (office) and remote (home) work conditions.

## 1 Introduction

Vision is one of the five basic senses of humans. In a world based on visual perception, the ability to see becomes a key aspect of daily life, accompanying individuals at every stage. It is essential from birth to old age. For a newborn, vision helps in recognizing the mother, and as the child grows and develops, it aids in maintaining balance and learning to walk. During the preschool and school years, it supports reading, in adulthood—professional work, and in old age—maintaining independence (1).

**Figure 1:**
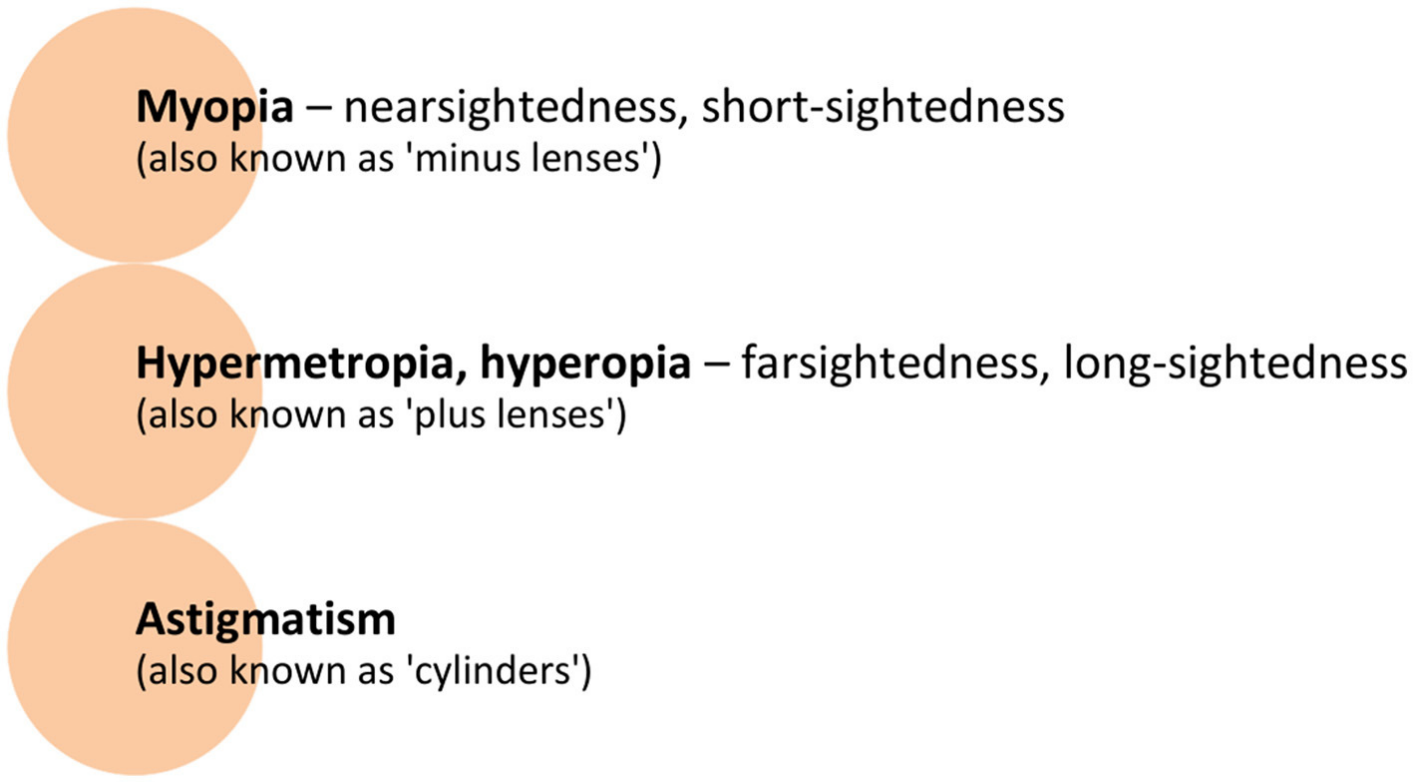
Common refractive errors of the eye (6–10). Source: Author's own elaboration.

Unfortunately, vision is also at risk of dysfunction and defects. Eye diseases are disorders that cause disturbances or loss of the ability to see (1, 2). These can be either congenital or acquired. An example of a congenital eye defect is persistent hyperplastic primary vitreous (PHPV), also known as persistent fetal vasculature syndrome (PFVS), while acquired defects include age-related macular degeneration (AMD) (3, 4). Some eye conditions, due to their high incidence and association with aging and environmental factors, are considered civilization diseases, such as cataracts and glaucoma (5). Eye diseases are sometimes associated with other conditions, such as diabetic retinopathy in diabetes (2). In recent years, refractive errors, which are problems with visual acuity caused by improper focusing of light on the retina, resulting in blurred vision, have gained increased attention as common vision defects. The most common refractive errors are shown in Figure 1 (6–10).

Furthermore, conjunctivitis is a commonly occurring eye disease with a diverse etiology. The underlying cause can be allergic, viral, or bacterial (11). Another common eye condition is dry eye disease (DED), also known as dry eye syndrome (DES), Sicca syndrome, or simply dry eyes. DED is characterized by an instability of the tear film, which may result from insufficient tear production or from poor tear film quality, leading to increased evaporation of tears (12).

**Figure 2:**
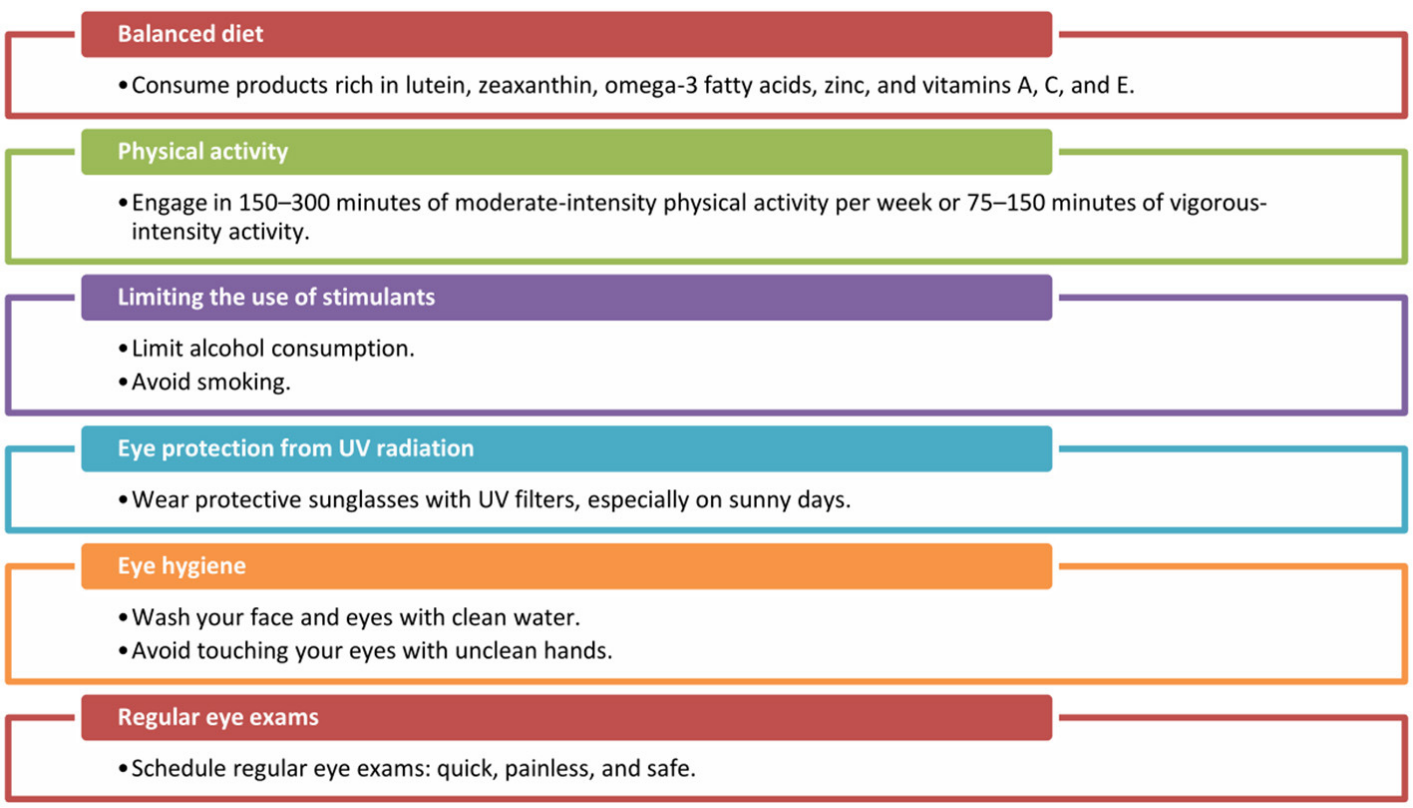
Lifestyle modifications reducing the risk of eye diseases (2, 13, 18–20). Source: Author's own elaboration.

Eye diseases and vision disorders are quite common. According to the World Health Organization (WHO), at least 2.2 billion people worldwide have some form of visual impairment, and among them, at least 1 billion have an impairment that could have been prevented or is yet to be effectively treated (1, 2). In Poland, nearly 50% of people aged 16-54 experience vision problems (2). Eye diseases are more prevalent in low- and middle-income countries, rural communities, ethnic minorities, and the older adult (1, 13). Additionally, the modern lifestyle, including poor diet, lack of physical activity, office work, and the use of artificial lighting and screen devices, is contributing to the rising incidence of eye diseases in younger age groups (2). In today's digital age, the use of screen devices has become widespread, both during the day and at night. Moreover, for most adults, work is intrinsically linked to the use of monitors (14). According to Article 6733 of the Polish Labor Code, every employee is entitled to work remotely for up to 24 days per year (15). Remote work requires employees to use monitors while performing their tasks and often leads to prolonged screen time (16). However, extended use of screens can result in eye strain and the development of common refractive errors (e.g., myopia) or dry eye syndrome. Given the increasing prevalence of monitor use in daily life and the rising number of visual impairments, this has become an emerging public health concern in Polish society (6, 17). Recent forecasts indicate that the demand for ophthalmic care will increase in the coming years, due to the growing global population, aging societies, and changes in lifestyle (1). Eye disease prevention includes all actions and measures aimed at preventing various ophthalmic conditions and maintaining good eye health throughout life. Its goal is to minimize the risk of eye diseases, prevent vision loss, and promote overall eye health. The basic preventive measures for eye diseases are shown in Figure 2 (2, 13, 18–20). An important aspect of eye disease prevention also includes proper workplace hygiene when using monitors, as well as public and healthcare staff education (2, 3).

Treatment of eye diseases depends on the type of condition, its stage of progression, and the individual needs of the patient (3). In the case of glaucoma, laser therapy or surgical procedures may be used (21). Refractive errors are corrected with glasses or contact lenses (22). Treatment of ocular surface diseases may involve the use of eye drops, ointments, or oral medications, depending on the type and cause of the problem. For example, treatment of conjunctivitis may include the use of antibacterial or antiviral medications (23, 24). In some cases, regular supplementation of vitamins and minerals is enough to support eye health (18, 19).

Treatment of eye diseases should always be conducted by a qualified eye specialist, such as an ophthalmologist, who is usually responsible for initial diagnosis and management eye conditions. In some cases, the ophthalmologist may refer patients to other specialists for further investigations or treatment if necessary. Early and accurate diagnosis helps minimize disease progression and ensures the implementation of effective treatment. Following medical recommendations and attending regular check-ups allow for monitoring the progress of treatment and any potential side effects of therapy (3).

The aim of the study was to assess the current, self-reported vision health of working individuals and evaluate their awareness and attitudes toward eye disease prevention (both in office and remote work environments).

## 2 Materials and methods

### 2.1 Study design

This study used a cross-sectional design to survey the current vision health, and evaluate awareness and attitudes toward eye disease prevention among a sample of working participants.

In the third quarter (Q) of 2023, the number of employed individuals aged 15–89 years amounted to 16,873,000 people (56.8% of the population in this age group), and in the fourth quarter, it was 17,323,000 people (57.1%). In both quarters, there were more men (54.0% in Q3 and 53.8% in Q4), and more people working in urban areas (59.0% in Q3 and 60.5% in Q4). The employment rate was higher among men (64.0%) than among women (50.1% in Q3 and 50.6% in Q4). A higher employment rate was observed in rural areas in Q3 (57.4%) compared to urban areas (56.3%), while in Q4, the higher employment rate was in urban areas (57.2%) compared to rural areas (56.8%) (25, 26). The demographic profile of the study participants seems to closely resemble that of the overall working population in Poland.

The survey was conducted between September 27, 2023, and December 1, 2023. The questionnaire described in the Section 2.3 was posted on social media platform, specifically on forum dedicated to surveys for research purposes and academic theses. It was designed for respondents who found the survey topic relevant to complete it independently. This publicly available forum encourages individuals to share their opinions and support cross-sectional studies. The selection of forum was based on keywords entered into search engines, such as 'surveys,' 'surveys for theses,' and 'surveys for academic research'. This forum collectively had ~4.5 thousand participants. When forming the group, both the reach of its impact and the level of difficulty in joining were considered, aiming to ensure diversity among participants and include individuals from various geographical locations. Consequently, groups of a “private” nature were excluded from the selection process. Completing the electronic questionnaire was entirely voluntary and anonymous. Each respondent was informed of these conditions, including the full anonymity of their participation and the voluntary nature of the study (Appendix 1). Respondents could withdraw from the study at any time. Due to the nature of the study, approval from the bioethics committee was not required.

### 2.2 Study group

The primary criterion for inclusion in the analysis was participants' current employment status on the labor market at the time of the survey. Eligibility for the study group required being actively employed (regardless of age, gender, place of residence, or education level). It should be noted that the sample selection was purposeful in accordance with the study's objectives to include participants with relevant characteristics.

### 2.3 Research tool

The data in this article come from a study conducted for the purposes of a master's thesis. The proprietary survey questionnaire contained 83 closed-ended questions: 65 single-choice questions and 18 multiple-choice questions (Appendix 1). This article includes the results obtained from the analysis of 34 selected questions (26 single-choice questions and 8 multiple-choice questions) from the four sections of the questionnaire. The selection of 34 questions was based on their direct relevance to the research aims and hypotheses formulated for this article. The questions were thematically divided into the following sections:

- Demographic information: gender, age, place of residence, education (4 questions);
- Questions related to work/profession (2 questions);
- Questions regarding visual health (9 questions);
- Questions concerning the awareness of eye disease prevention (19 questions), 16 of which were assessed using an original awareness scoring system.

The survey was conducted using a Google Forms (RRID:SCR_023174) template.

To assess the clarity and comprehensibility of the questionnaire items, a pilot study was conducted on a group of 15 working adults. The pilot testing took place in person, allowing for the observation of respondents' reactions and the collection of their feedback regarding the content and structure of the questions. Participants were asked to complete the questionnaire and indicate any difficulties in understanding specific questions or response options. Based on the feedback received, minor editorial adjustments were made to improve the clarity and reliability of the tool. The questionnaire was developed based on a review of the literature on eye health prevention and after consultations with specialists in ophthalmology and public health, which allowed its content to be tailored to the specific characteristics of the target population.

To assess the level of awareness regarding eye disease prevention, 16 questions in the questionnaire were assigned a predetermined number of points. A correct answer to each question earned 1 point, with a maximum score of 38 points (11 points from 11 single-choice questions and 27 points from 5 multiple-choice questions). In the case of multiple-choice questions where both a correct and incorrect answer were selected, 0 points were awarded. To interpret the results, a five-point grading scale was used: 0–14 points—insufficient (ins) awareness level; 15–20 points—acceptable (acc) awareness level; 21–26 points—satisfactory (sat) awareness level; 27–32 points—good (good) awareness level; 33–38 points—very good (vg) awareness level. The scoring of the questions was done using a Microsoft Excel (RRID:SCR_016137) 2019 spreadsheet.

### 2.4 Statistical analysis

The statistical analysis was conducted using GraphPad Prism (RRID:SCR_002798) 8.3.0 software. For sample sizes >5, the non-parametric Chi-square (χ^2^) test of independence was applied. For samples of 5 or fewer, Fisher's exact test, a non-parametric method, was used. The Shapiro-Wilk test was employed to assess normality of distribution. If results indicated normality, Bartlett's test for homogeneity of variances and the parametric Analysis of Variance (ANOVA) were applied. In cases where the distribution was non-normal or mixed, the non-parametric Kruskal-Wallis test was used. A significance level of *p* < 0.05 was set.

## 3 Results

### 3.1 Characteristics of the study group

The study included 251 participants, comprising 124 women (49.40%) and 127 men (50.60%). The median and mode age range of the study group was 40–49 years. The majority of participants were from rural areas (25.10%) and held higher (44.22%) or secondary (33.86%) education. The largest group of respondents worked in office jobs (48.21%) or a combination of office and physical work (32.67%). The largest proportion of participants had 11–20 years of work experience (27.09%) or more than 20 years (25.50%). Detailed data are presented in Table 1.

**Table 1:** Characteristics of the study group.

**Total population (*****N*** = **251)**		
**Demographic information**	***n***	**%**
**Gender**		
° Female	124	49.40
° Male	127	50.60
**Age**		
° Under 20 years	12	4.78
° 20–29 years	51	20.32
° 30–39 years	49	19.52
° 40–49 years	81	32.27
° 50–59 years	53	21.12
° 60 years and over	5	1.99
**Place of residence**		
° Village (rural status)	63	25.10
° Town with up to 20,000 inhabitants (urban status)	40	15.94
° Town with 20,000–50,000 inhabitants (urban status)	38	15.14
° Town with 50,000–100,000 inhabitants (urban status)	31	12.35
° Town with 100,000–200,000 inhabitants (urban status)	33	13.15
° Town with 200,000–500,000 inhabitants (urban status)	12	4.78
° City with over 500,000 inhabitants (urban status)	34	13.55
**Education level**		
° Primary	9	3.59
° Junior high school	7	2.79
° Vocational	39	15.54
° Secondary	85	33.86
° Higher	111	44.22
**Type of work**		
° Physical work	48	19.12
° Office work	121	48.21
° Physical and office work	82	32.67
**Years of experience in the current type of work**		
° Less than a year	12	4.78
° 1–2 years	23	9.16
° 3–5 years	33	13.15
° 6–10 years	51	20.32
° 11–20 years	68	27.09
° More than 20 years	64	25.50

*N*, population size; *n*, sample size.

### 3.2 Vision health: determinants and perceptions

A majority of the respondents (62.55%) were found to have acquired vision impairments, which may include refractive errors such as hyperopia (37.85%) and myopia (21.51%). Vision defects were notably more common in women than in men, with a statistically significant difference between the groups (*p* = 0.0002). Additional data on vision health are presented in Table 2.

**Table 2:** Eye condition of the studied population.

**Studied characteristic**	**Women**		**Men**		**Total**		***p***
	***n***	**%**	***n***	**%**	***n***	**%**	
**Type of diagnosed eye defect**							
° Congenital	18	14.52	7	5.51	25	9.96	0.0015
° Acquired	83	66.94	74	58.27	157	62.55	
° Not diagnosed	23	18.55	46	36.22	69	27.49	
**Diagnosed refractive error of the eye**							
° Myopia (“nearsightedness”)	32	25.81	22	17.32	54	21.51	0.0002
° Hyperopia (“farsightedness”)	43	34.68	52	40.94	95	37.85	
° Astigmatism (“cylindrical”)	25	20.16	7	5.51	32	12.75	
° None of the above	24	19.35	46	36.22	70	27.89	
**Ophthalmic procedures performed**							
° Yes	22	17.74	17	13.39	39	15.54	0.3858
° No	102	82.26	110	86.61	212	84.46	
**Taking medications/supplements to improve vision**							
° Regularly	32	25.81	38	29.92	70	27.89	0.4784
° Occasionally	48	38.71	40	31.50	88	35.06	
° Never	44	35.48	49	38.58	93	37.05	
**Ophthalmologist appointments**							
° Several times a year	6	4.84	3	2.36	9	3.59	0.2139
° Once a year	48	38.71	49	38.58	97	38.65	
° Once every 2 years	16	12.90	16	12.60	32	12.75	
° Less than once every 2 years	15	12.10	7	5.51	22	8.76	
° Only when experiencing eye problems	35	28.23	42	33.07	77	30.68	
° I do not visit an ophthalmologist	4	3.23	10	7.87	14	5.58	
**Total**	124	100	127	100	251	100	-

*n*, sample size; *p*, statistical significance.

A significant majority of the respondents (84.46%) had never undergone any ophthalmic surgeries. A total of 88 participants (35.06%) reported occasionally taking medications or supplements to improve their vision health. A notable share of respondents (38.65%) visited an ophthalmologist once a year, while 30.68% stated that they only visited an eye doctor when experiencing problems with their eyes. In all three cases, comparisons between women and men revealed no statistically significant differences—*p* > 0.05 (Table 2).

Refractive eye disorders were most common among individuals aged 40–49 years (25.11%), with hyperopia being the most prevalent condition in this age group (20.72%). Myopia and astigmatism were most commonly observed in individuals aged 20–29 years (6.37 and 4.78%, respectively) and 30–39 years (5.58 and 5.18%, respectively). No statistically significant differences were observed between the age groups (Table 3). Refractive eye disorders were more common among individuals working in office jobs (42.23%) or in office jobs combined with physical work (22.71%; Table 4). Myopia was most common among individuals who had been working in the respective job for over 20 years (5.18%) and for 6–10 years (4.78%). Hyperopia was most frequently observed in individuals with 11 or more years of work experience (18.73%). Statistical analysis using Bartlett's test showed that the variances between the compared groups were not homogeneous. Furthermore, the ANOVA test did not provide evidence of statistically significant differences between these groups (Table 5).

**Table 3:** Refractive error of the eye and age.

**Studied characteristic**	**Under 20 years**		**20–29 years**		**30–39 years**		**40–49 years**		**50–59 years**		**60 years and over**	
	***n***	**%**	***n***	**%**	***n***	**%**	***n***	**%**	***n***	**%**	***n***	**%**
Ns	3	1.20	16	6.37	14	5.58	8	3.19	13	5.18	-	-
Fs	-	-	2	0.80	4	1.59	52	20.72	36	14.34	1	0.40
A	4	1.59	12	4.78	13	5.18	3	1.20	-	-	-	-
None	5	1.99	21	8.37	18	7.17	18	7.17	4	1.59	4	1.59
Kruskal-Wallis test = 3.120; *p* = 0.3736												

A, astigmatism; Fs, farsightedness; Ns, nearsightedness; *n*, sample size; *p*, statistical significance.

**Table 4:** Refractive error of the eye and type of work.

**Studied characteristic**	**Physical work**				**Office work**				**Physical and office work**			
	***n***		**%**		***n***		**%**		***n***		**%**	
Ns	8	18	3.19	7.17	24	106	9.56	42.23	22	57	8.76	22.71
Fs	9		3.59		61		24.30		25		9.96	
A	1		0.40		21		8.37		10		3.98	
None	30		11.95		15		5.98		25		9.96	
χ^2^ = 50.15; *df* = 6; *p* < 0.0001												

A, astigmatism; Fs, farsightedness; Ns, nearsightedness; df, degrees of freedom; *n*, sample size; *p*, statistical significance.

**Table 5:** Refractive error of the eye and years of experience in the current type of work.

**Studied characteristic**	**Less than a year**		**1–2 years**		**3–5 years**		**6–10 years**		**11–20 years**		**More than 20 years**	
	***n***	**%**	***n***	**%**	***n***	**%**	***n***	**%**	***n***	**%**	***n***	**%**
Ns	4	1.59	7	2.79	11	4.38	12	4.78	7	2.79	13	5.18
Fs	-	-	1	0.40	6	2.39	7	2.79	47	18.73	34	13.55
A	4	1.59	3	1.20	7	2.79	12	4.78	5	1.99	1	0.40
None	4	1.59	12	4.78	9	3.59	20	7.97	9	3.59	16	6.37
Bartlett's test = 19.56; *p* = 0.0002 → ANOVA: *F* = 1.047; *R*^2^ = 0.1358; *p* = 0.3933												

A, astigmatism; F, the ratio of between-group variance to within-group variance; Fs, farsightedness; Ns, nearsightedness; R^2^, coefficient of determination; *n*, sample size; *p*, statistical significance.

Taking medications or supplements to improve vision “regularly” was more common in two age groups: 40–49 years (13.94%) and 50–59 years (10.36%). On the other hand, using products to support vision quality “occasionally” began even before the age of 20 years in some cases (1.59%; Table 6).

**Table 6:** Use of medications/supplements for vision improvement and age.

**Studied characteristic**	**Under 20 years**		**20–29 years**		**30–39 years**		**40–49 years**		**50–59 years**		**60 years and over**	
	***n***	**%**	***n***	**%**	***n***	**%**	***n***	**%**	***n***	**%**	***n***	**%**
Regularly	1	0.40	1	0.40	4	1.59	35	13.94	26	10.36	3	1.20
Occasionally	4	1.59	24	9.56	20	7.97	24	9.56	16	6.37	-	-
Never	7	2.79	26	10.36	25	9.96	22	8.76	11	4.38	2	0.80
Kruskal-Wallis test = 0.5549; *p* = 0.7758												

*n*, sample size; *p*, statistical significance.

The largest percentage of study participants (*n* = 89; 35.46%) reported that confirming a vision impairment changed their attitude toward eye care and led to better eye hygiene. Among the most common concerns expressed by respondents regarding eye diseases/impairments were fears of being unable to work and the potential financial problems associated with it (*n* = 137; 54.58%), as well as concerns about losing independence and becoming dependent on the help of others (*n* = 106; 42.23%).

### 3.3 Assessment of awareness of eye disease prevention

Table 7 presents the participants' subjective assessments of their awareness regarding eye disease prevention. More than half of the respondents rated their awareness as basic (58.87% of women and 57.48% of men).

**Table 7:** Subjective assessment of respondents' awareness toward the prevention of eye diseases.

**Assessment**	**Women**		**Men**		**Total**	
	***n***	**%**	***n***	**%**	***n***	**%**
Very low	6	4.84	12	9.45	18	7.17
Low	14	11.29	13	10.24	27	10.76
Basic	73	58.87	73	57.48	146	58.17
High	25	20.16	17	13.39	42	16.73
Very high	6	4.84	12	9.45	18	7.17
Total	124	100	127	100	251	100
χ^2^ = 5.526; df = 4; p = 0.2375						

df, degrees of freedom; *n*, sample size; *p*, statistical significance.

Table 8 presents the aggregated results of participants' awareness about eye disease prevention. Thirty-four individuals (13.55%) scored up to and including 14 points, thus receiving the lowest rating. The majority of participants demonstrated a satisfactory (32.27%) and good (30.28%) level of awareness. Only 25 individuals (9.96%) received an excellent rating. The lowest score was 4 points, which was achieved by a man, while the highest score (37 points) was obtained by a woman. The results of the test were not influenced by the participants' gender.

**Table 8:** Level of awareness toward the prevention of eye diseases among respondents.

**Level of awareness**	**Women**		**Men**		**Total**	
	***n***	**%**	***n***	**%**	***n***	**%**
Insufficient (0–14 pts)	15	12.10	19	14.96	34	13.55
Acceptable (15–20 pts)	11	8.87	24	18.90	35	13.94
Satisfactory (21–26 pts)	42	33.87	39	30.71	81	32.27
Good (27–32 pts)	40	32.26	36	28.35	76	30.28
Very good (33–38 pts)	16	12.90	9	7.09	25	9.96
Total	124	100	127	100	251	100
χ^2^ = 7.546; *df* = 4; *p* = 0.1097						

df, degrees of freedom; *n*, sample size; *p*, statistical significance; pts, points.

Among the respondents with the lowest rating, individuals with secondary education predominated (4.38%). Most participants with secondary or higher education achieved satisfactory or good ratings. The highest level of awareness, resulting in an excellent rating, was observed in only 5.98% of individuals with higher education. The arithmetic mean of the scores showed that individuals with higher education achieved the best results among all education levels (25.55). Bartlett's test indicated a lack of homogeneity in the variances across the compared groups (*p* = 0.0111). Detailed data are presented in Table 9.

**Table 9:** Level of awareness toward the prevention of eye diseases and education level.

**Level of awareness**	**Primary**		**Junior high school**		**Vocational**	**Secondary**		**Higher**	
	***n***	**%**	***n***	**%**	***n***	**%**	***n***	**%**	***n***	**%**
Ins	3	1.20	5	1.99	7	2.79	11	4.38	8	3.19
Acc	1	0.40	1	0.40	7	2.79	13	5.18	13	5.18
Sat	2	0.80	-	-	11	4.38	33	13.15	35	13.94
Good	2	0.80	1	0.40	10	3.98	23	9.16	40	15.94
Vg	1	0.40	-	-	4	1.59	5	1.99	15	5.98
Avg.	21.00		13.71		22.74		23.02		25.55	
Bartlett's test = 13.05; *p* = 0.0111 → ANOVA: *F* = 1.085; *R*^2^ = 0.1783; *p* = 0.3906										

Avg., arithmetic mean; F, the ratio of between-group variance to within-group variance; R^2^, coefficient of determination; *n*, sample size; *p*, statistical significance.

A notable share of individuals who received a satisfactory rating were engaged in office work (16.73%) or a combination of physical and office work (12.75%). Employees who received a good rating were similarly often involved in office-related tasks (14.74%). The highest rating was achieved by office workers (4.38%). No statistically significant differences were observed between the compared groups (*p* > 0.05; Table 10).

**Table 10:** Level of awareness toward the prevention of eye diseases and type of work.

**Level of awareness**	**Physical work**		**Office work**		**Physical and office work**	
	***n***	**%**	***n***	**%**	***n***	**%**
Ins	11	4.38	12	4.78	11	4.38
Acc	5	1.99	19	7.57	11	4.38
Sat	7	2.79	42	16.73	32	12.75
Good	20	7.97	37	14.74	19	7.57
Vg	5	1.99	11	4.38	9	3.59
χ^2^ = 14.65; *df* = 8; *p* = 0.0663						

df, degrees of freedom; *n*, sample size; *p*, statistical significance.

**Figure 3:**
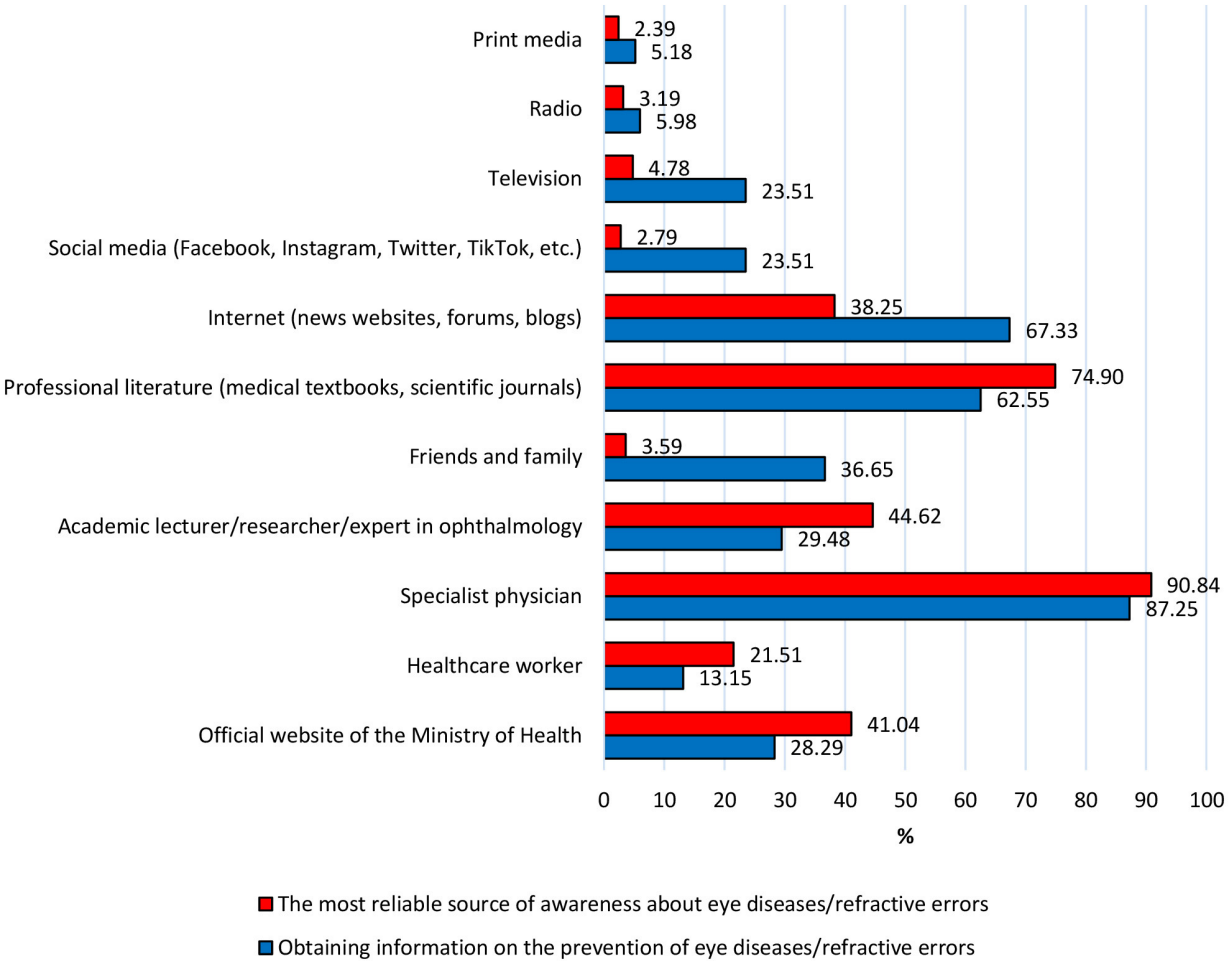
The most reliable sources of information on eye diseases/refractive errors, compared to sources on prevention, according to respondents. Source: Author's own elaboration.

The respondents identified a specialist doctor (*n* = 228; 90.84%) and professional literature, such as medical books and scientific journals (*n* = 188; 74.90%), as the most reliable sources of information about eye diseases/impairments. Similarly, the most common sources of awareness about eye disease prevention were a specialist doctor (*n* = 219; 87.25%), ranked first, followed by the Internet, including informational websites, social media forums, and blogs (*n* = 169; 67.33%). In this context, professional literature ranked third (*n* = 157; 62.55%). The data are summarized in Figure 3. Typically, all the above-mentioned sources were indicated simultaneously by individuals with secondary or higher education.

## 4 Discussion

The population of working individuals is an extremely important group within society. Their role and impact on the functioning and development of the economy, including sectors such as healthcare, make them a key and fundamental element of a dynamically evolving society. Good health promotes the productivity of the workforce. High rates of sickness absenteeism in the workplace can negatively affect performance, leading to increased costs related to employee replacements and placing a strain on healthcare services. This necessitates action from personnel management, as well as efforts to improve employee health and wellbeing. Therefore, maintaining good health among the working population at the highest possible level is a critical aspect of economic efficiency and the sustainable development of society (27).

Working individuals belong to occupational groups that are particularly exposed to acquired eye diseases. Employment involving computer use is an integral part of daily professional life in various sectors, especially in offices, information technology, marketing, finance, and many other industries. Awareness of the potential risks associated with this type of work, along with knowledge of methods to mitigate them, enables the prevention of negative health outcomes (28, 29). Therefore, the aim of the study was to examine the current eye health condition of a group of professionally active individuals, as well as their awareness of eye disease prevention in both stationary and remote work environments.

The conducted study revealed that over half of employed individuals had acquired visual impairments. Among these, farsightedness was the most prevalent (37.85% of the study group), followed by nearsightedness (21.51%). Statistical analysis confirmed that women were significantly more likely to suffer from refractive eye defects. For comparison, Kozłowski et al. (30) found that residents of Lublin most frequently reported farsightedness (73.8%), while nearsightedness was observed in 7.6% of these residents. Similarly, Krakowiak et al. (31) reported that 54.40% of Warsaw residents had one of the following vision impairments: nearsightedness, farsightedness, astigmatism, or strabismus. Henrykowska et al. (32) also demonstrated that farsightedness and nearsightedness were the most common ocular conditions among employees of the Polish Post. It is important to highlight that ocular conditions pose a concern not only for working individuals but also frequently affect students, adolescents, and preschool-aged children. Ciecierska et al. (33) reported that nearsightedness was present in 71.5% of students identified with visual impairments.

It is worth noting that 84.46% of respondents in the author's study reported never having undergone any ophthalmic surgeries. However, 88 individuals (35.06%) admitted to occasionally using medications or supplements to improve their eye health. Additionally, only 38.65% regularly visited an ophthalmologist once a year. A concerning observation is that 30.68% of respondents consulted a specialist only when specific eye problems arose. This suggests a lack of regular preventive check-ups, which could lead to delayed detection of potential eye issues and less effective prevention. Similar findings were reported by Krakowiak et al. (31), where 48% of respondents visited an ophthalmologist only when experiencing eye problems, while just 12% underwent regular annual eye examinations. Kozłowski et al. (30) also found that 61.4% of Lublin residents consulted an ophthalmologist only in cases of vision difficulties. By contrast, findings from another study conducted in Poland indicated that 7.1% of adults had never had their eyes examined (34). A similar tendency has been observed in the UK, where almost 14 million people do not undergo regular eye tests, despite these being essential health assessments (35). A survey conducted by the Guide Dogs' Communications Team, involving 2,000 participants and 20 questions about eye health, found that 8% of respondents—rising to 10% among men—had never received any form of vision screening or eye care appointment (36). Respondents were more likely to seek ophthalmological consultations for their children. According to Wisłocka et al. (37), preventive eye consultations were reported for only 16.75% of children. The conducted study did not reveal any correlation between refractive errors and age, refractive errors and work experience, or the frequency of using medications/supplements for eye health and age. The analysis indicates that refractive eye errors may be associated with the type of work performed, with the highest prevalence observed among office workers. This is likely attributable to the nature of their work, which predominantly involves focusing on close objects and tasks. However, as the study is cross-sectional, it cannot determine causality or the onset of refractive errors. The workers may have had pre-existing conditions prior to their employment, so the study does not directly attribute their eye defects to their occupation.

The respondents appear reluctant to engage in eye health prevention. Our results indicate that only the presence of a visual impairment can be a significant factor influencing increased attention to eye care (prompting greater care of eye hygiene), while also triggering concerns related to professional life and independence. Greater awareness of these concerns could help direct preventive and educational efforts to improve eye health and support individuals with visual impairments in various aspects of life. Both women and men equally rated their awareness as basic. The equality in the subjective assessment of awareness levels between women and men may stem from similar access to informational and educational resources, which results in a comparable level of awareness evaluation.

The eye disease prevention awareness test revealed variations in the level of awareness among respondents based on their education level and occupation. A small group of individuals scored below the minimum threshold, indicating insufficient awareness. Most of these individuals had lower levels of education, suggesting the presence of a group of individuals who may require special and more targeted attention regarding eye disease prevention. On a positive note, the predominant group of the adult working study population demonstrated either satisfactory or good awareness. This group was predominantly composed of individuals with secondary or higher education, many of whom were employed in office jobs or in manual work with office-related tasks. Workplaces that offer structured tasks or knowledge-sharing opportunities might play a role in spreading health-related information. This is a positive sign that both educational background and the certain types of work performed may influence awareness about eye disease prevention, but it also underscores that much remains to be done in terms of raising awareness on this issue. A particularly concerning observation is that only a small percentage of individuals with higher education (5.98%) received the highest rating of “excellent” awareness, indicating that even those with advanced education may not fully understand or prioritize eye health, highlighting a gap that could be addressed through more comprehensive and accessible health education programs. Therefore, the results highlight the need to tailor and align educational efforts to different educational groups, especially among those with secondary education, while emphasizing the importance of equal access to information about eye disease prevention across all social groups. While education and work environment seem to positively influence awareness about eye disease prevention, there is a clear need for broader and more effective initiatives, such as campaigns, to ensure even highly educated individuals are well-informed about maintaining eye health. Insufficient knowledge about eye disease prevention is also pointed out by the study conducted by Kozłowski et al. (30). Studies by Kamińska et al. also indicate the low level of awareness about eye diseases among Poles and the need to improve this awareness. Only 10% of respondents reported a very good or rather good level of knowledge about eye diseases, while 46.3% stated that their level of knowledge was rather poor or very poor. Most respondents had heard of common eye diseases, such as cataracts (83.6%), glaucoma (80.7%), and conjunctivitis (74.3%). However, only 50% of respondents were aware of a condition particularly relevant for individuals working mainly with screens, namely dry eye syndrome (38). In contrast, the study by Alshammari *et al*. found that 66% of respondents were aware of dry eyes. The difference in awareness levels between Saudi Arabia (66%) and Poland (50%) could be attributed to several factors, such as public health campaigns, work and lifestyle differences (e.g., extensive screen use in air-conditioned environments), and healthcare access and communication. This highlights the importance of tailored approaches that consider regional and environmental factors to improve awareness in different populations (39). Studies conducted in Nepal, Germany, and Saudi Arabia have also revealed significant gaps in awareness and knowledge about eye health and common eye diseases. Research across different populations underscores the importance of enhancing awareness and knowledge, which should be integrated into educational interventions aimed at the public. These efforts are crucial for preventing eye diseases, promoting early treatment, and ensuring access to ophthalmic care (39–41).

According to the results of the author's study, specialists are regarded as the most reliable source of awareness about eye diseases/defects and their prevention by the vast majority of respondents. Professional literature, such as medical books and scientific journals, is also trusted. This confirms the trust placed in medical specialists as authorities in the field of eye health and highlights the importance of their medical advice. Information from professional scientific publications is also highly valued by the respondents. In contrast, the high ranking of the Internet (news portals, social forums, blogs) as a popular source of information on prevention reflects the increasing significance of access to online medical information. Research by Wisłocka et al., conducted among children aged 2–10, shows that for 28.9% of parents, a doctor is the primary source of information regarding prevention and alarming symptoms of eye diseases or defects in young children. The second most common source was the Internet, at 27.5%. The press was also relatively popular, with 18.2% (37). In the author's study, individuals with secondary and higher education often identified specialists, professional literature, and the Internet as reliable sources of knowledge. This suggests that people with higher education tend to use a variety of sources, both traditional and digital, to obtain comprehensive information. However, it is important that effective educational strategies on eye health consider the diversity of information sources, while also fostering trust in medical specialists and professional scientific publications.

Both the results of the conducted studies and the analysis of available scientific publications clearly demonstrate that the respondents possess some awareness of eye disease prevention, but their attitudes toward preventive measures were insufficient. This highlights the need for educational initiatives in the field of eye disease prevention, particularly among people who are professionally active and especially those exposed to prolonged screen time. Health education is an economically accessible and often effective strategy for preventing eye diseases. It is important to expand awareness and shape appropriate attitudes starting at the school education level and throughout vocational training. The need for education in this area also becomes increasingly important as the population ages. Equally crucial is raising awareness of eye disease prevention among older individuals. By 2030, one in six people globally will be aged 60 or older, with this demographic increasing from 1 billion in 2020 to 1.4 billion. By 2050, the number will reach 2.1 billion, effectively doubling (42). This rapid growth highlights the importance of targeted health education and prevention measures, as aging is closely linked to a higher risk of chronic conditions, including eye diseases. Furthermore, an aging society will see a growing number of individuals who may become partially or fully dependent on others for daily assistance. Proactively addressing these challenges is essential to reduce the burden on caregivers and healthcare systems while ensuring a better quality of life for those who are no longer self-sufficient. Additionally, it is crucial to systematically monitor employees' awareness levels, which can be achieved by emphasizing the importance of regular preventive screenings. Professionally active employees should have a comprehensive understanding of potential risks and effective prevention methods. Given the concerning projections that predict no improvement in this area, the topic remains crucial and warrants further discussion.

This study has several limitations. We acknowledge that the sample is not representative of the entire population, which may have contributed to the lack of statistical significance. The insufficient sample size may mean that the results do not reflect differences in preferences or behaviors between the studied groups, making it harder to identify clear patterns or trends. Therefore, the results require further research with a larger sample to confirm or expand upon the findings. One of the main limitations of this study is the potential for selection bias due to the self-recruitment nature of the online survey. Individuals experiencing vision problems may have been more likely to participate, given the topic's relevance to their personal health. Therefore, the high prevalence of vision impairments observed in the sample should be interpreted with caution, as it may reflect selection bias rather than true population-level trends. This study represents an initial step in exploring the topic, and due to its multi-phase design, future research may allow for broader and more representative data collection. Another limitation relates to the classification of refractive errors in the questionnaire. Participants were asked to select a refractive condition (e.g., myopia, hyperopia, astigmatism) based on self-perception, without clinical diagnosis or clear definitions provided. As a result, some respondents—particularly older individuals—may have misclassified presbyopia as hyperopia. Additionally, some participants may not have been familiar with the term “presbyopia”, or they may not have been aware that they had developed this age-related condition. It is also important to note that not all aging individuals experience presbyopia. These factors may have contributed to an overestimation of hyperopia prevalence in the sample and should be considered when interpreting the results. A limitation of the study that could affect the validity of the survey results is its length, which may lead to respondent fatigue due to the large number of questions. This, in turn, might result in random or careless answers, without any attempt to read or understand the questions. Nevertheless, we would like to highlight the critical importance of eye health prevention in a world where our vision is increasingly strained, particularly in an environment dominated by screens, digital devices, and continuous exposure to visual stimuli. Future research should focus on developing effective strategies to enhance public awareness and improve attitudes toward eye health prevention, particularly in the context of modern-day challenges. This should include evaluating the effectiveness of educational campaigns, exploring the role of technological interventions in promoting preventive practices, and examining how demographic factors, such as age, education, and occupation, impact attitudes and behaviors related to eye health.

## 5 Conclusions

The eye health condition of the surveyed population does not meet expectations and is not satisfactory. At the same time, the awareness level of the surveyed group of professionally active individuals regarding eye disease prevention seems to be insufficient.

Although these findings cannot be generalized to the broader population due to the limitations of the study, the data presented suggest areas that may require particular attention and intervention. Efforts could be made to disseminate awareness about eye disease prevention in both stationary and remote work environments within Polish society. It is essential to consider the key role of technology and emphasize the need to promote information about the safe use of digital devices, often called digital hygiene. It also seems important to take action to promote more professional sources of knowledge.

## Data Availability

The raw data supporting the conclusions of this article will be made available by the authors, without undue reservation.
