# Supplementary material for: Attitudes and awareness of professionally active people on eye diseases prevention—a descriptive cross-sectional survey
Source: Front Public Health. 2025 Jun 3;13:1553361. doi: 10.3389/fpubh.2025.1553361 (PMC12170685; doi:10.3389/fpubh.2025.1553361)
Supplement: Supplementary file 1 [file Data_Sheet_1.pdf]

Supplementary material

Appendix 1

Assessment of the awareness and attitudes among working individuals towards the prevention of eye diseases

Dear Sir/Madam,

I am a second-year Master's student in Public Health at the Medical University of Lodz. As part of my Master's thesis, I am conducting a study on the "**Assessment of the awareness and attitudes among working individuals towards the prevention of eye diseases**". This survey is addressed to employed individuals, and I kindly invite you to participate.

Completing the questionnaire is entirely voluntary and anonymous. You can stop filling out the survey at any stage, which will be considered as withdrawing from the study. Due to the nature of the study, approval from the bioethics committee was not required.

For some questions, you can select one or more answers. Please provide honest responses. By submitting the completed questionnaire, you consent to the use of the information solely for scientific purposes (Act on the Protection of Personal Data of 29 August 1997, Journal of Laws No. 133, item 883). The data collected will be analyzed and presented as aggregated statistics. The results of this survey will be used exclusively for scientific purposes.

Thank you for your time and for completing the questionnaire! 😊

Karina Mierczak

INFORMED CONSENT TO PARTICIPATE IN THE STUDY

At the beginning, I will ask you to select your answer regarding participation in the study. Please choose the appropriate option:

- Yes, I agree to participate in the study, I am over 18 years old, and I confirm that I will complete the survey.
- No, I do not agree to participate in this study, I am under 18 years old, and I will not complete the survey.

Remember, you can stop filling out the survey at any time and withdraw from the study. If you have any questions, feel free to contact me: **karina.mierczak@stud.umed.lodz.pl**

DEMOGRAPHIC INFORMATION

Gender:

- Female
- Male

Age:

- Under 20
- 20-29
- 30-39
- 40-49
- 50-59
- 60 and over

Place of residence:

- Village
- Town with up to 20,000 inhabitants
- Town with 20,000 to 50,000 inhabitants
- Town with 50,000 to 100,000 inhabitants
- Town with 100,000 to 200,000 inhabitants
- Town with 200,000 to 500,000 inhabitants
- City with over 500,000 inhabitants

Education level:

- Primary
- Junior high school
- Vocational
- Secondary
- Higher

QUESTIONS RELATED TO WORK/PROFESSION

What type of work do you do?

- Physical work
- Office work
- Physical and office work

How long have you been doing the selected type of work?

- Less than a year
- 1-2 years
- 3-5 years
- 6-10 years
- 11-20 years
- More than 20 years

QUESTIONS REGARDING VISUAL HEALTH

Has an eye disease/refractive error been diagnosed in you?

- Yes
- No

What type of eye defect has been diagnosed in you?

- Congenital
- Acquired
- No eye defect has been diagnosed in me

What type of refractive error of the eye do you have?

- Myopia (“nearsightedness”)
- Hyperopia (“farsightedness”)
- Astigmatism (“cylindrical”)
- None of the above

Have you undergone any ophthalmic procedures?

- Yes
- No

Do you take any medications or supplements to improve your vision?

- Yes, regularly
- Yes, occasionally
- No, never

How often do you visit an ophthalmologist?

- Several times a year
- Once a year
- Once every two years
- Less than once every two years
- Only when I have eye problems
- I do not visit an ophthalmologist

Has the confirmation of an eye disease/refractive error changed your attitude toward eye health prevention?

- Yes, I understood the seriousness of the situation and started taking care of my eye hygiene
- Yes, my concern increased that my eyesight may worsen further
- No, nothing has changed
- No eye disease/refractive error has been confirmed in me

Do you feel afraid of eye disease/refractive error?

- Definitely not
- Rather not
- Rather yes
- Definitely yes

Why do you feel afraid of eye disease/refractive error?
(You can select more than one answer.)

- I fear the reaction of my family, friends, and society
- I fear being excluded from society
- I fear being unable to work and the potential financial problems that could arise
- I fear becoming dependent on others for help

- I fear the severe form of the disease/error
- I fear death
- I do not fear eye disease/refractive error

QUESTIONS CONCERNING THE AWARENESS OF EYE DISEASE PREVENTION

How would you rate your awareness of the causes of eye diseases/refractive errors?

- Very low
- Low
- Basic
- High
- Very high

What behaviors or symptoms, in your opinion, might indicate serious eye diseases/refractive errors and require an ophthalmological consultation?
(You can select more than one answer.)

- Blurry near vision, poor sharpness
- Blurred vision at a distance
- Double vision
- Excessive blinking
- Squinting
- Tearing
- Redness of the eyes
- Eye burning
- Dry eyes
- Light sensitivity
- Eye pain
- Eye injury
- Dizziness
- Fatigue

Do you think that the occurrence of eye diseases/refractive errors could be related to lifestyle?

- Yes, lifestyle has a very significant impact on the occurrence of eye diseases/refractive errors
- No, lifestyle has no impact on the occurrence of eye diseases/refractive errors
- I don’t know, I haven’t considered this

Do you think the occurrence of eye diseases/refractive errors can be linked to the presence of other diseases, such as diabetes?

- Yes, the occurrence of eye diseases/refractive errors can be linked to the presence of other diseases
- No, the occurrence of eye diseases/refractive errors is not linked to the presence of other diseases
- I don’t know, I haven’t considered this

In your opinion, what can reduce the risk of eye diseases/refractive errors?
(You can select more than one answer.)

- Healthy nutrition
- Physical activity
- Elimination of stimulants
- Eye protection (e.g., using protective eye drops, contact lenses)
- Regular eye exams

Which vitamin, in your opinion, may contribute to weakened vision?
(You can select more than one answer.)

- Vitamin A

- Vitamin C
- Vitamin E
- I don't know

Which substances, in your opinion, contribute to improving vision?
(You can select more than one answer.)

- Lutein
- Spirulina
- Zeaxanthin
- Piperine
- I don't know

What, in your opinion, is the simplest and most commonly used method to diagnose and determine the severity of an eye disease?

- Ophthalmological examination
- Cardiac examination
- Tomographic examination
- Otolaryngological examination
- Blood test

Do you think that working with screen devices contributes to eye discomfort?

- Yes
- No
- I don't know

According to you, who is more likely to experience eye discomfort?

- People working at positions with a single monitor
- People working at positions with multiple monitors

What do you think is the main cause of dry eye syndrome (Sicca syndrome)?

- Insufficient tear secretion
- Abnormal shape of the pupil
- Lack of rhodopsin
- All of the above

How would you describe computer vision syndrome, in your opinion?

- A modern health issue
- An infectious condition
- Incurable

What are the typical symptoms of computer vision syndrome, in your opinion?

- Tear secretion disorders
- Sweat secretion disorders
- Hormone secretion disorders
- Tear and sweat secretion disorders
- Tear, sweat, and hormone secretion disorders

How long after turning on an LED monitor, in your opinion, is the electrostatic radiation most intense?

- 5 minutes after turning on the monitor
- 15 minutes after turning on the monitor
- 35 minutes after turning on the monitor
- 55 minutes after turning on the monitor

What, in your opinion, is the most appropriate type of lighting for a workspace with a monitor?

- Natural light (sunlight)
- Artificial light
- A combination of natural (sunlight) and artificial light

What light do you think all monitors emit?

- Yellow light
- Green light
- Red light
- Blue light
- Purple light

According to you, what can blue light emitted by monitors contribute to?
(You can select more than one answer.)

- Retinal photochemical damage
- Decreased melatonin secretion
- Increased alertness, including reduced sleepiness
- Decreased alertness, including increased sleepiness

What, in your opinion, is the most reliable source of awareness about eye diseases/refractive errors?
(You can select more than one answer.)

- Official website of the Ministry of Health
- Healthcare worker
- Specialist physician
- Academic lecturer/researcher/expert in ophthalmology
- Friends and family
- Professional literature (medical textbooks, scientific journals)
- Internet (news websites, forums, blogs)
- Social media (Facebook, Instagram, Twitter, TikTok, etc.)
- Television
- Radio
- Print media
- None of the above
- I have never heard of eye diseases

Where do you obtain information on the prevention of eye diseases/refractive errors?
(You can select more than one answer.)

- Official website of the Ministry of Health
- Healthcare worker
- Specialist physician
- Academic lecturer/researcher/expert in ophthalmology
- Friends and family
- Professional literature (medical textbooks, scientific journals)
- Internet (news websites, forums, blogs)
- Social media (Facebook, Instagram, Twitter, TikTok, etc.)
- Television
- Radio
- Print media
- None of the above
- I have never heard of eye disease prevention
